# Risk factors for pelvic organ prolapse and its recurrence: a systematic review

**DOI:** 10.1007/s00192-015-2695-8

**Published:** 2015-05-13

**Authors:** Tineke F. M. Vergeldt, Mirjam Weemhoff, Joanna IntHout, Kirsten B. Kluivers

**Affiliations:** Department of Obstetrics and Gynecology, Radboud University Medical Center, P.O. Box 9101, 6500 HB Nijmegen, The Netherlands; Department of Obstetrics and Gynecology, Atrium Medical Center Parkstad, Heerlen, The Netherlands; Department for Health Evidence, Radboud University Medical Center, Nijmegen, The Netherlands

**Keywords:** Pelvic organ prolapse, Recurrence, Risk factors

## Abstract

**Introduction and hypothesis:**

Pelvic organ prolapse (POP) is a common condition with multifactorial etiology. The purpose of this systematic review was to provide an overview of literature on risk factors for POP and POP recurrence.

**Methods:**

PubMed and Embase were searched with “pelvic organ prolapse” combined with “recurrence” and combined with “risk factors,” with Medical Subject Headings and Thesaurus terms and text words variations until 4 August 2014, without language or publication date restrictions. Only cohort or cross-sectional studies carried out in western developed countries containing multivariate analyses and with a definition of POP based on anatomical references were included. POP recurrence had to be defined as anatomical recurrence after native tissue repair without mesh. Follow-up after surgery should have been at least 1 year. Articles were excluded if POP was not a separate entity or if it was unclear whether the outcome was primary POP or recurrence.

**Results:**

PubMed and Embase revealed 2,988 and 4,449 articles respectively. After preselection, 534 articles were independently evaluated by two researchers, of which 15 met the selection criteria. In 10 articles on primary POP, 30 risk factors were investigated. Parity, vaginal delivery, age, and body mass index (BMI) were significantly associated in at least two articles. In 5 articles on POP recurrence, 29 risk factors were investigated. Only preoperative stage was significantly associated in at least two articles.

**Conclusion:**

Parity, vaginal delivery, age, and BMI are risk factors for POP and preoperative stage is a risk factor for POP recurrence.

## Introduction

Female pelvic organ prolapse (POP) is a common condition that is multifactorial in etiology [1]. It is likely that combinations of anatomical, physiological, genetic, lifestyle, and reproductive factors interact throughout a woman’s lifespan to contribute to pelvic floor dysfunction [2]. The factors causing POP development vary from patient to patient [3]. Unraveling the complex causal network of genetic factors, birth-induced injury, connective tissue aging, lifestyle, and co-morbid factors is challenging [2].

While two thirds of parous women have anatomical evidence of POP [4], the majority of these women are asymptomatic [5]. It has been reported that in a general population 40 % of women aged between 45 and 85 years have an objective POP on examination, but only 12 % of these women are symptomatic [6]. Women with symptomatic disorders suffer physical and emotional distress [7]. It has a great negative impact on women’s social, physical, and psychological well-being [8]. As the general population ages, pelvic floor dysfunction will become increasingly burdensome in terms of reduced quality of life, workforce productivity, and cost to both the individual and the health care system as a whole [9].

The lifetime risk of surgery for POP in the general female population is 11.1 % [10]. Surgery for POP is known to have a high reoperation rate [10]. The identification of risk factors for POP development and its recurrence therefore appears crucial for the best management of women with this condition to provide proper preoperative counseling or modulate patients’ expectations and tailor surgical treatment [11].

An overview of the literature on risk factors for POP and its recurrence after native tissue repair would help to build a risk model to identify low- and high-risk women. The purpose of this systematic review was to provide an overview of the published literature on risk factors for the development of POP and its recurrence after native tissue repair.

## Materials and methods

The primary investigator (TFMV) and a clinical librarian searched the electronic databases PubMed and Embase with the search terms “pelvic organ prolapse” in combination with “recurrence,” and “pelvic organ prolapse” in combination with “risk factors” from inception until 4 August 2014. To capture all relevant articles on this subject, Medical Subject Headings (MeSH) and Thesaurus terms and text words with different word variations were used. Restrictions on publication date or language were not applied. The searches are depicted in the Appendices A1 and A2.

At first, all studies were evaluated by title. Of the papers available, those titles were selected that might contain information about risk factors for primary POP or POP recurrence.

After this preselection, two researchers (TFMV and MW) independently evaluated all studies by abstract. If there was disagreement, full-text articles were evaluated. If the full text was unavailable, authors were contacted to obtain the article. Abstracts were included in case they reported on clinical studies on the etiology or risk factors for primary POP or POP recurrence. Letters, commentaries, and editorial notes were excluded. The full text of the articles included was assessed using an in- and exclusion form. Cohort studies or cross-sectional studies carried out in western developed countries were included. The definition of POP had to be based on anatomical references such as the hymenal remnants or the Pelvic Organ Prolapse Quantification (POPQ) system stage 2. POP recurrence had to be defined as anatomical recurrence after native tissue repair (i.e., without the use of mesh materials and follow-up after surgery should at least be 1 year. Furthermore, articles had to contain a multivariate analysis. Articles were excluded if they did not study POP as a separate entity (but investigated pelvic floor dysfunction in general), if it was unclear whether the outcome was a primary POP or a POP recurrence (e.g., after hysterectomy) and in case POP recurrence was studied after mesh augmentation. If there were more publications using the same study population, only the most recent study was included. If there was disagreement on the in- or exclusion of an article after discussion between the two observers, the decision was made by asking the opinion of one of the other researchers in the research group (KBK).

A manual search of the references of each selected article was performed to further identify studies not captured by the online search, but potentially relevant for this review.

After the final selection, data were extracted on study design, the aim of the study, sample size, the study population, the definition of outcome, the risk factors investigated, and the results of the multivariate analysis. If multiple analyses were performed with different definitions of POP, data regarding the definition “POPQ stage 2 or more” or closest to this definition, were extracted.

*P* values <0.05 were considered statistically significant. Only risk factors that were significantly associated with POP or POP recurrence in the multivariate analysis in at least two studies, were defined as confirmed risk factors.

## Results

**Fig. 1:**
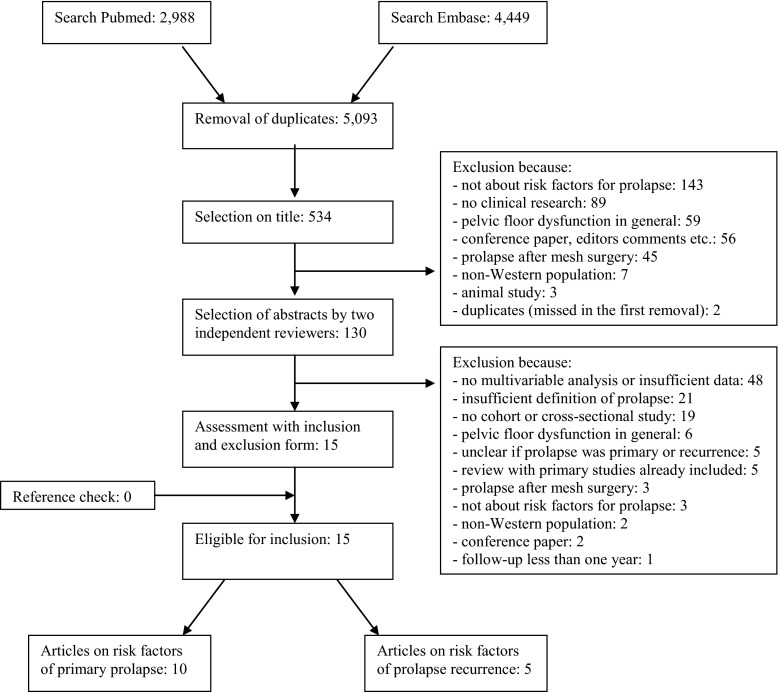
Flow diagram of the selection process

The PubMed search and the Embase search revealed 2,988 and 4,449 articles respectively. After elimination of duplicates, 5,093 articles were evaluated by title and/or abstract. Full texts of 130 articles were assessed using the in- and exclusion form, of which 15 articles met the selection criteria. No additional studies were identified by cross-checking reference lists. Of the 15 articles included in this systematic review, 10 investigated risk factors for primary POP and 5 articles investigated risk factors for POP recurrence after surgery. Figure 1 shows the flow diagram of the selection process.

### Risk factors for primary POP

The articles investigating potential risk factors for primary POP are listed in Table 1. Of the 10 articles included, 7 were cross-sectional studies and 3 were prospective cohort studies. Overall, the quality of the studies included was assessed as adequate: all studies had clear participant recruitment and selection criteria; the outcome and covariates were clearly defined; the results were well presented; sample sizes were sufficient for the number of predictor variables examined (i.e., more than 10 events per candidate variable) [12]. In 3 studies it was explicitly described that the examining physician was blinded to other data, such as a questionnaire or ultrasound findings [6, 13, 14].

**Table 1:** The articles on primary prolapse included in the study

Reference	Study type	*N*/*n*	Inclusion criteria	Risk factors
Progetto Menopausa Italia Study Group [20]	Cross-sectional study	21,449/410	Nonhysterectomized women around menopause attending an outpatient menopause clinic for general counselling about menopause	BMI, delivery mode, age, parity, smoking, education, birth weight, age at menarche, age at menopause
Nygaard et al [15]	Cross-sectional study	270/173	Nonhysterectomized women enrolled in the WHI Hormone Replacement Therapy clinical randomized trial	BMI, delivery mode, age, smoking, hormone replacement therapy, education, birth weight, waist circumference, occupation, physical activity, family history, age at first and last delivery, pulmonary disease, previous hernia surgery
Swift et al [13]	Cross-sectional study	1,004/218	Women older than 18 years of age presenting for routine gynecological health care	BMI, delivery mode, age, parity, smoking, ethnicity, hormone replacement therapy, birth weight, constipation, occupation, hysterectomy status, menopausal status, chronic illness, income, gravidity
Whitcomb et al [16]	Cross-sectional study	1,137/762	Women between 40 and 69 years of age who, since age 18 years, had been members of the Kaiser Permanente Medical Care Program of Northern California	BMI, age, parity, ethnicity, education, diabetes
Slieker-Ten Hove et al [6]	Cross-sectional study	649/227	A general population of women aged 45 to 85 years	BMI, age, parity, smoking, menopausal status, education, physical activity, family history, urinary incontinence, prolapse during pregnancy
Handa et al [17]	Prospective cohort study	1,011/75	Women between 15 and 50 years of age giving birth to their first child 5 to 10 years before enrolment	Delivery mode
Kudish et al [21]	Prospective cohort study	12,650/2,266	Nonhysterectomized postmenopausal women enrolled in the WHI Estrogen plus Progestin Clinical Trial	BMI, age, parity, smoking, ethnicity, hormone replacement therapy, waist circumference, constipation, physical activity, pulmonary disease, urinary incontinence
Dietz et al [14]	Cross-sectional study	605/NA^a^	Women without previous incontinence or prolapse surgery with symptoms of pelvic floor dysfunction, with data of four-dimensional ultrasound	Levator avulsion, hiatal area on Valsalva
Glazener et al [18]	Prospective cohort study	762/182	Women who delivered over a 12-month period in three maternity units	BMI, delivery mode, parity, age at first birth
Yeniel et al [19]	Cross-sectional study	1,964/155	Women without previous prolapse surgery with benign gynecological disorders	BMI, delivery mode, smoking, menopausal status

*N*/*n* number of women included in the study who underwent physical examination/number of women with pelvic organ prolapse,* BMI* Body Mass Index in kg/m^2^, WHI Women’s Health Initiative, NA not available

^a^Number of women categorized by type of prolapse: 222 women with cystocele, 159 women with rectocele, 40 women with apical prolapse

The 10 articles included enrolled a total of 41,501 women. POP was defined as POPQ stage 2 or more in 4 studies [6, 14–16], as the most dependent point of the vaginal wall to or beyond the hymenal remnants in 3 studies [17–19], as degree 2 or 3 of the Baden–Walker classification system in 1 study [20], as the most dependent point of the vaginal wall to the introitus or outside of the vagina (according to the Women’s Health Initiative classification system) in 1 study [21], and as the most dependent point of the vaginal wall –0.5 cm above the hymenal remnants in 1 study [13].

In the 10 articles, 30 potential risk factors were investigated, of which 17 were significantly associated with primary POP at least once in the multivariate analysis. Obstetric factors are represented in Table 2. Other potential risk factors are shown in Table 3.

**Table 2:** Obstetric risk factors for primary prolapse

Risk factor	Times investigated	Times statistically significant	*N*	Definition	Adjusted OR^a^ (95 % CI)	Reference
Delivery mode	7	5	21,449	≥1 cesarean vs no cesarean	OR 0.6 (0.4-1.0)	[20]
			270	Per 1 vaginal delivery	OR 1.6 (1.0-2.5)*	[15]
				No vaginal deliveries vs 1 or 2	OR 0.0 (0.0–0.4)*	
				No vaginal deliveries vs 3 or 4	OR 0.1 (0.0–0.5)*	
				No vaginal deliveries vs ≥5	OR 0.1 (0.0–0.6)*	
				3 or 4 vaginal deliveries vs 1 or 2	OR 0.7 (0.3–1.7)	
				≥5 vaginal deliveries vs 1 or 2	OR 1.2 (0.4–3.4)	
			1,004	Per 1 vaginal delivery	OR 1.1 (0.9–1.4)	[13]
			1,137	Cesarean only vs nulliparous	PR 1.1 (1.0–1.2)*	[16]
				≥1 vaginal delivery vs nulliparous	PR 1.1 (1.1–1.2)*	
			1,011	All cesarean before full dilation vs all cesarean before labor	RR 0.5 (0.1–2.3)	[17]
				≥1 cesarean after full dilation vs all cesarean before labor	RR 0.7 (0.2–3.1)	
				Spontaneous vaginal births vs all cesarean before labor	RR 5.6 (2.2–14.7)*	
				≥1 operative vaginal birth vs all cesarean before labor	RR 7.5 (2.7–20.9)*	
			726	Cesarean only vs spontaneous vaginal delivery only	OR 0.1 (0.0–0.4)*	[18]
				≥1 forceps delivery vs spontaneous vaginal delivery only	OR 0.6 (0.4–1.0)*	
				≥1 vacuum extraction, no forceps vs spontaneous vaginal delivery only	OR 0.7 (0.4–1.4)	
				Vaginal and caesarean deliveries vs spontaneous vaginal delivery only	OR 0.5 (0.2–1.0)*	
			1,964	Vaginal delivery vs nulliparous	OR 2.9 (1.2–7.2)*	[19]
				Cesarean vs nulliparous	OR 0.3 (0.0–2.5)	
Parity	6	4	21,449	1 vs 0	OR 3.1 (1.5–6.4)*	[20]
				2 vs 0	OR 3.4 (1.7–6.7)*	
				≥3 vs 0	OR 4.6 (2.3–9.1)*	
			1,004	Per 1	OR 1.1 (0.7–1.7)	[13]
			649	1 vs 0	OR 0.4 (0.2–1.2)	[6]
				2 vs 0	OR 1.6 (0.9–2.7)	
				≥3 vs 0	OR 1.5 (0.9–2.8)	
			12,650	1 vs 0	HR 2.4 (1.7–3.6)*	[21]
				2 vs 0	HR 3.5 (2.5–4.9)*	
				3 vs 0	HR 3.9 (2.8–5.4)*	
				4 vs 0	HR 5.1 (3.7–7.1)*	
				≥5 vs 0	HR 5.9 (4.2–8.1)*	
			726	2 vs 1	OR 3.3 (1.5–7.3)*	[18]
				3 vs 1	OR 3.9 (1.7–9.2)*	
				≥4 vs 1	OR 5.2 (2.0–13.4)*	
			1,964	Per 1	OR 1.2 (1.1–1.4)*	[19]
Birth weight	3	1	21,449	>4,500 g vs ≤4,500 g	OR 1.3 (0.9–1.7)	[20]
			270	>3,690 g vs ≤3,690 g	NS^b^	[15]
			1,004	Per 10 ounces	OR 1.1 (1.0–1.2)*	[13]
Age at first delivery	2	1	270	<20 vs 20–24 vs ≥25	NS^b^	[15]
			726	25–29 vs ≤24	OR 1.5 (0.9–2.3)	[18]
				30–34 vs ≤24	OR 2.5 (1.5–4.2)*	
				≥35 vs ≤24	OR 3.1 (1.4–6.6)*	
Age at last delivery	1	0	270	≤29 vs 30–34 vs ≥35	NS^b^	[15]
Gravidity	1	0	1,004	Per 1	OR 0.9 (0.7–1.2)	[13]

*N* number of participants, OR odds ratio, 95 % CI  95 % confidence interval, PR prevalence ratio, RR risk ratio, HR hazard ratio, NS not statistically significant

*Statistically significant association (*p* < 0.05)

^a^In some studies PR, RR or HR was used

^b^No other data in article

**Table 3:** Non-obstetric risk factors for primary prolapse

Risk factor	Times investigated	Times significantly different	*N*	Definition	Adjusted OR^a^ (95 % CI)	Reference
Lifestyle factors						
BMI	8	5	21,449	23.8–27.2 vs <23.8	OR 1.6 (1.2–2.2)*	[20]
				>27.2 vs <23.8	OR 1.8 (1.3–2.4)*	
			270	<27 vs ≥27	NS^b^	[15]
			1,004	25–30 vs <25	OR 2.5 (1.2–5.4)*	[13]
				>30 vs <25	OR 2.6 (1.2–5.4)*	
			1,137	25–30 vs <25	PR 1.1 (1.0–1.1)*	[16]
				≥30 vs <20	PR 1.1 (1.0–1.1)*	
			649	Per kg/m^2^	NS^b,c^	[6]
			12,650	25–30 vs <25	HR 1.3 (1.1–1.4)*	[21]
				≥30 vs <25	HR 1.3 (1.1–1.5)*	
			726	<18.5 vs 18.5–24.9	OR 1.2 (0.3–5.0)	[18]
				25–29.9 vs 18.5–24.9	OR 1.3 (0.9–2.0)	
				≥30 vs 18.5–24.9	OR 1.5 (0.9–2.4)	
			1,964	Per kg/m^2^	OR 1.0 (0.9–1.0)*	[19]
Smoking	6	3	21,449	<10 vs no	OR 1.6 (1.0–2.6)	[20]
				10–20 vs no	OR 1.1 (0.6–2.1)	
				>20 vs no	OR 1.3 (0.7–2.4)	
			270	Unknown	NS^b,c^	[15]
			1,004	Ever vs never	OR 1.2 (0.6–2.4)	[13]
				Current vs never	OR 0.9 (0.3–2.5)	
			649	Current vs no	OR 0.5 (0.3–0.8)*	[6]
			12,650	Past vs never	HR 0.8 (0.7–0.8)*	[21]
				Current vs never	HR 0.5 (0.4–0.7)*	
			1,964	Yes vs no	OR 0.6 (0.3–0.9)*	[19]
HRT	3	1	270	Unknown	NS^b,c^	[15]
			1,004	Ever vs never	OR 1.0 (0.6–1.7)	[13]
			12,650	E + P treatment vs placebo	HR 1.1 (1.0–1.3)*	[21]
				Past hormone use vs never	HR 1.1 (1.0–1.2)	
				Current hormone use vs never	HR 1.2 (1.0–1.5)	
Physical activity	3	0	270	Mild vs moderate vs strenuous	NS^b,c^	[15]
			649	Current heavy work vs no	OR 1.3 (0.9–2.0)	[6]
				Past heavy work vs no	NS^b,c^	
			12,650	Unknown	HR 1.0 (1.0–1.0)	[21]
Waist circumference	2	1	270	<88 cm vs ≥88 cm	NS^b^	[15]
			12,650	>88 cm vs <88 cm	HR 1.2 (1.0–1.4)*	[21]
Unmodifiable factors						
Age	6	4	21,449	52–55 vs ≤51	OR 1.5 (1.1–2.0)*	[20]
				≥56 vs ≤51	OR 2.6 (2.0–3.4)*	
			270	≥68 vs <68	NS^b,c^	[15]
			1,004	Per 10 years	OR 1.4 (1.1–1.8)*	[13]
			1,137	Per 10 years	PR 1.0 (1.0–1.1)*	[16]
			649	Per 1 year	NS^b,c^	[6]
			12,650	Per 1 year	HR 1.0 (1.0–1.0)*	[21]
Ethnicity	3	2	1,004	Black vs white	OR 1.2 (0.4–3.3)	[13]
				Hispanic vs white	OR 4.3 (1.8–10.2)*	
				Other vs white	OR 2.4 (0.5–12.1)	
			1,137	White vs African–American	PR 1.0 (1.0–1.1)	[16]
				Asian vs African–American	PR 1.0 (1.0–1.1)	
				Latina/other vs African–American	PR 1.0 (1.0–1.1)	
			12,650	Black vs white	HR 0.5 (0.4–0.7)*	[21]
				Hispanic vs white	HR 0.9 (0.7–1.1)	
Menopausal status	3	1	1,004	No vs yes	OR 0.6 (0.4–1.1)^c^	[13]
			649	Yes vs no	OR 1.3 (0.9–1.9)	[6]
			1,964	Yes vs no	OR 5.2 (3.4–8.0)*	[19]
Family history	2	0	270	Family with prolapse/UI surgery	NS^b,c^	[15]
			649	Mother with prolapse vs no	OR 1.6 (1.0–2.4)	[6]
Age at menopause	1	0	21,449	49–51 vs <48	OR 0.9 (0.7–1.3)	[20]
				≥52 vs <48	OR 1.1 (0.8–1.5)	
Age at menarche	1	0	21,449	12–13 vs <11	OR 0.8 (0.6–1.0)	[20]
				≥14 vs <11	OR 1.0 (0.8–1.3)	
Comorbidity						
Urinary incontinence	2	1	649	UI surgery vs no	OR 2.2 (0.9–5.4)	[6]
			12,650	Stress UI vs never	HR 1.1 (1.0–1.3)	[21]
				Urge UI vs never	HR 1.3 (1.1–1.5)*	
				Mixed UI vs never	HR 1.2 (1.0–1.5)*	
				Other UI vs never	HR 1.0 (0.8–1.4)	
Pulmonary disease	2	0	270	Asthma yes vs no	NS^b,c^	[15]
			12,650	Asthma	HR 1.0 (0.8–1.2)	[21]
				Emphysema	HR 1.2 (0.9–1.6)	
Constipation	2	0	1,004	Yes vs no	NS^c,d^	[13]
			12,650	Moderate/severe vs no	HR 1.0 (0.8–1.2)	[21]
Diabetes	1	1	1,137	Yes vs no	PR 1.1 (1.1–1.1)*	[16]
Chronic illness	1	0	1,004	Any vs none	OR 1.1 (0.5–2.1)^c^	[13]
Hysterectomy status	1	0	1,004	Yes vs no	OR 1.1 (0.7–1.6)^c^	[13]
Previous hernia surgery	1	0	270	Yes vs no	NS^b,c^	[15]
POP in pregnancy	1	0	649	Yes vs no	OR 1.4 (1.0–2.1)	[6]
Social factors						
Education	4	2	21,449	Intermediate school vs elementary	OR 0.6 (0.5–0.8)*	[20]
				High school/university vs elementary	OR 0.6 (0.4–0.8)*	
			270	≤High school vs > high school	OR 2.2 (1.1–4.2)*	[15]
			1,137	≥College vs < college	PR 1.0 (1.0–1.1)	[16]
			649	Intermediate school vs unknown	OR 0.7 (0.4–1.1)	[6]
Occupation	2	0	270	Previous employment history	NS^b,c^	[15]
			1,004	Labor vs nonlabor	OR 1.2 (0.6–2.3)^c^	[13]
Income	1	1	1,004	Medium vs high	OR 0.3 (0.1–0.8)*	[13]
				Low vs high	OR 1.4 (0.5–3.9)	
Pelvic floor factors						
Levator defect	1	1	605	Unilateral vs no avulsion	OR 2.8 (1.4–5.4)*	[14]
				Bilateral vs no avulsion	OR 4.0 (1.8–9.1)*	
Hiatus genitalis	1	1	605	Hiatal area on Valsalva per cm^2^	OR 1.1 (1.1–1.1)*	[14]

*HRT* hormone replacement therapy,* E + P * estrogen plus progesterone, * UI* urinary incontinence,* POP* pelvic organ prolapse

*Statistically significant association (*p* < 0.05)

^a^In some studies hazard ratio or prevalence ratio was used

^b^No other data in article

^c^Data of univariate analysis, not in multivariate analysis

^d^Described in article twice with different results, both not significant

### Risk factors for POP recurrence

The articles investigating potential risk factors for prolapse recurrence are listed in Table 4. Of the 5 articles included, 3 were prospective cohort studies and 2 were retrospective cohort studies. Overall, the quality of the studies included was assessed as adequate: all studies had clear participant recruitment and selection criteria; the outcome and covariates were clearly defined; results were well presented; median follow-up after surgery was between 1 and 12 years. However, selective loss to follow-up could not be excluded in 1 study, in which less than half of the women included had attended the follow-up visit and no comparisons were reported between women attending the follow-up visit and women not attending the follow-up visit [22]. In 4 out of 5 studies the number of risk factors evaluated was higher than generally advised (i.e., 10 events per candidate variable) [12, 23–26]. For example, 1 study had 36 events (i.e., prolapse recurrence) and assessed 10 candidate variables [23], and another study had 42 events and assessed 12 candidate variables [24]. In 1 study it was explicitly described that the examining physician was blinded to other data, such as a questionnaire or ultrasound findings [25].

**Table 4:** Articles on prolapse recurrence included

Reference	Study type	*N*/*n*	Inclusion criteria	Follow-up	Risk factors
Tegerstedt and Hammarstrom [26]	Retrospective cohort study	128/56	Women who had prolapse surgery (Manchester procedure, anterior colporrhaphy, posterior colporrhaphy, cervix amputation, vaginal hysterectomy, enterocele repair, abdominal vaginosacropexy or combinations)	10–12 years	Age, preoperative stage, BMI, pulmonary disease, smoking, urinary incontinence, complicated delivery, previous pelvic floor surgery, heavy lifting, incomplete emptying of bladder, constipation, fecal incontinence, surgeon’s experience
Whiteside et al [22]	Prospective cohort study	176/102	Women who underwent anterior colporrhaphy, with or without hysterectomy, posterior colporrhaphy, bladder neck plication, vaginal vault suspension, enterocele repair, culdoplasty, bladder neck suspension or retropubic paravaginal defect repair	1 year	Age, preoperative stage, hysterectomy status, number of sites involved, urinary incontinence, previous prolapse surgery, menopausal status, diabetes, site of most advanced preoperative prolapse, previous incontinence surgery
Diez-Itza et al [24]	Retrospective cohort study	134/42	Women who had vaginal hysterectomy, anterior colporrhaphy or posterior colporrhaphy for prolapse	5 years	Age, preoperative stage, BMI, constipation, pulmonary disease, parity, family history, surgeon’s experience, weight, abdominal hernias, intense physical exercise, levator muscle contraction
Salvatore et al [23]	Prospective cohort study	360/36	Women who underwent prolapse surgery without using grafts (vaginal hysterectomy, and/or anterior colporrhaphy and/or posterior colporrhaphy)	26 months	Age, preoperative stage, BMI, constipation, hysterectomy status, pulmonary disease, parity, genital hiatus, menopausal status, birth weight
Weemhoff et al [25]	Prospective cohort study	156/80	Women who underwent anterior colporrhaphy, with or without hysterectomy, posterior colporrhaphy or sacrospinous fixation	2 years	Age, preoperative stage, BMI, constipation, parity, number of sites involved, family history, concomitant surgery, previous prolapse surgery, complicated delivery, levator defect

*N*/*n* number of women included in the study who underwent physical examination/number of women with pelvic organ prolapse recurrence

The 5 articles included enrolled a total of 954 women of which 316 with POP recurrence. POP recurrence was defined as POPQ stage 2 or more in all studies.

In the 5 articles, 29 potential risk factors were investigated, of which 8 were significantly associated at least once with POP recurrence after surgery in the multivariate analysis (Table 5).

**Table 5:** Risk factors for prolapse recurrence

Risk factor	Times investigated	Times statistically significant	*N*	Definition	Adjusted OR (95 % CI)	Reference
Obstetric factors						
Parity	3	0	134	0 vs ≥1	NS^a^	[24]
			360	Per 1	NS^a^	[23]
			156	Per 1	OR 0.9 (0.7–1.2)^b^	[25]
Complicated delivery	2	0	128	Yes vs no	OR 1.4 (0.9–1.9)^b^	[26]
			156	Assisted vs no	OR 0.8 (0.3–2.1)^b^	[25]
Birth weight	1	0	360	>4,000 g vs ≤4,000 g	OR 1.8 (0.9–3.6)^b^	[23]
Age at last delivery	1	0	134	Per 1	NS^a^	[24]
Lifestyle factors						
BMI	4	0	128	>25 vs ≤25	OR 1.2 (0.9–1.8)^b^	[26]
			134	Per kg/m^2^	NS^a^	[24]
			360	>30 vs ≤30	OR 1.2 (0.5–2.8)^b^	[23]
			156	Per kg/m^2^	OR 1.0 (0.9–1.1)^b^	[25]
Weight	1	1	134	>65 vs ≤65	OR 4.0 (1.6–9.6)*	[24]
Intense physical exercise	1	0	134	Yes vs no	NS^a^	[24]
Heavy lifting	1	0	128	Yes vs no	OR 1.1 (0.7–1.6)^b^	[26]
Smoking	1	0	128	Yes vs no	OR 1.4 (0.8–2.5)^b^	[26]
Unmodifiable factors						
Age	5	2	128	>70 vs ≤70	NS^a^	[26]
			176	<50 vs 50–59 vs 60–69 vs ≥70 <60 vs ≥60	NS^a^ OR 3.2 (1.6–6.4)*	[22]
			134	<60 vs ≥60	OR 4.1 (1.6–10.4)*	[24]
			360	Age per year	NS^a^	[23]
			156	Age per year	NS^a^	[25]
Family history	2	1	134	Yes vs no	NS^a^	[24]
			156	Yes vs no	OR 2.4 (1.2–4.9)*	[25]
Menopausal status	2	0	176	Yes vs no	NS^a^	[22]
			360	Yes vs no	NS^a^	[23]
Comorbidity						
Constipation	4	0	128	Yes vs no	OR 1.1 (0.7–1.7)^b^	[26]
			134	Yes vs no	NS^a^	[24]
			360	Yes vs no	OR 0.6 (0.3–1.4)^b^	[23]
			156	Yes vs no	OR 1.0 (0.4–2.3)^b^	[25]
Previous pelvic floor surgery	3	1	128	Yes vs no	OR 1.8 (1.1–2.8)^b^*	[26]
			176	Yes vs no	NS^a^	[22]
			156	Yes vs no	OR 1.4 (0.5–4.0)^b^	[25]
Pulmonary disease	3	0	128	Yes vs no	OR 1.3 (0.7–2.4)^b^	[26]
			134	Yes vs no	NS^a^	[24]
			360	Yes vs no	OR 1.6 (0.7–3.8)^b^	[23]
Any incontinence preoperative	2	1	128	Yes vs no	OR 1.4 (1.0–2.1)^b^*	[26]
			176	Yes vs no	NS^a^	[22]
Previous hysterectomy	2	0	176	Yes vs no	NS^a^	[22]
			360	Yes vs no	OR 0.6 (0.3–1.2)^b^	[23]
Incomplete emptying of bladder	1	0	128	Yes vs no	OR 1.3 (0.9–1.9)^b^	[26]
Fecal incontinence	1	0	128	Yes vs no	NS^a^	[26]
Diabetes	1	0	176	Yes vs no	NS^a^	[22]
Abdominal hernias	1	0	134	Yes vs no	NS^a^	[24]
Surgical factors						
Preoperative stage	5	4	128	Stage IV vs < stage IV	OR 1.5 (0.9–2.4)	[26]
			176	Stage III or IV vs stage II	OR 2.7 (1.3–5.3)*	[22]
			134	Stage III or IV vs stage I or II	OR 3.9 (1.2–13.0)*	[24]
			360	Stage III or IV vs stage I or II	OR 2.4 (1.1–5.1)*	[23]
			156	Stage III or IV vs stage I or II	OR 2.0 (1.0–4.1)*	[25]
Surgeon’s experience	2	0	128	Senior vs no senior surgeon	OR 0.8 (0.5–1.3)^b^	[26]
			134	Junior vs no junior surgeon	NS^a^	[24]
Number. of sites involved preoperative	2	0	176	1 vs 2 vs 3	NS^a^	[22]
			156	2 vs 1 3 vs 1	OR 1.1 (0.5–2.5)^b^ OR 0.7 (0.3–1.8)^b^	[25]
Concomitant surgery	1	1	156	Sacrospinal fixation vs no	OR 6.5 (2.0–21.2)*	[25]
Pelvic floor factors						
Levator defect	1	1	156	Yes vs no	OR 2.3 (1.1–4.8)*	[25]
Hiatus genitalis	1	0	360	Unknown	OR 1.4 (0.5–2.3)^b^	[23]
Levator muscle contraction	1	0	134	Oxford scale <3 vs ≥3	NS^a^	[24]
Site of most advanced prolapse	1	0	176	Anterior vs apex vs posterior	NS^a^	[22]

*Statistically significant association (*p *< 0.05)

^a^No other data in article

^b^Data of univariate analysis, not in multivariate analysis

### Risk factors discussed by topic

#### Obstetric factors

Parity and vaginal delivery were frequently investigated and shown to be risk factors for primary POP [15, 16, 18–21], except in 2 studies [6, 13]. The association with cesarean delivery was less clear. While in 2 studies no association between cesarean delivery and primary POP was found [19, 20], 1 study showed that cesarean delivery was a risk factor when compared with nulliparous women [16], and 2 studies found that it was protective when compared with spontaneous or operative vaginal delivery [17, 18]. There was a trend toward an association between larger birth weight and primary POP, but only in 1 out of 3 studies was this statistically significant [13, 15, 20]. Higher age at first delivery was a risk factor in 1 study [18], but in another study no significant association was found [15]. Operative vaginal delivery, age at last delivery, and gravidity were investigated only once and no significant associations were found, except for forceps delivery, which was protective against primary POP when compared with spontaneous vaginal delivery only [13, 15, 18].

For POP recurrence, parity and complicated delivery were not significant risk factors [23–25]. This was in contrast with primary POP, for which parity was a risk factor. This phenomenon might be because in studies concerning POP recurrence, only women with a primary POP are included; therefore, this is a selected group of women. Birth weight and age at last delivery were only investigated once and no significant association was found [23, 24].

#### Lifestyle factors

Higher body mass index (BMI) as a categorical variable was a significant risk factor for primary POP [13, 16, 20, 21], except for the 2 studies with the smallest sample sizes [15, 18]. Two studies investigated BMI as a continuous variable, of which 1 found no association [6] and in contrast with the other studies, 1 found that a higher BMI was slightly protective [19]. Waist circumference and use of hormone replacement therapy were each only once significantly associated with primary POP; thus, no conclusion can be drawn [13, 15, 21]. The results for the relation between smoking and primary POP were inconsistent. One study showed a trend toward a positive association [20], while in 3 studies smoking was protective [6, 19, 21], and in 2 studies no association was found [13, 15]. One study argued that there might be an association between cigarette smoking and POP because smoking causes chronic respiratory diseases and higher abdominal pressure, but a negative association was found because smoking seemed to be linked to factors such as age and menopausal status [19]. This hypothesis was supported by the fact that in another study the seemingly protective effect disappeared in the multivariate analysis [13]. Physical activity was not a significant risk factor for primary POP [6, 15, 21].

Although higher BMI was a risk factor for primary POP, it was not a significant risk factor for POP recurrence [23–26]. Weight, intense physical exercise, heavy lifting, and smoking were examined only once and only weight was significantly associated with POP recurrence, but no firm conclusions can be drawn owing to a lack of confirmation [24, 26].

#### Unmodifiable factors

Age was a risk factor for primary POP [13, 16, 20, 21], except in the 2 smallest studies [6, 15]. The role of ethnicity remained unclear in relation to primary POP. In 1 study a higher risk in Hispanic women compared with white women was found, while in another study there was no significant association [13, 21]. Another study found a higher risk in white women compared with black women, while 2 other studies found no association [13, 16, 21]. Menopausal status showed a trend toward a positive association with primary POP, but in only 1 of the 3 studies was it a significant risk factor [6, 13, 19]. Family history was not a significant risk factor [6, 15]. Age at menopause and age at menarche were only examined once and showed no association [20].

Age as a risk factor for POP recurrence showed inconsistent results. In 2 studies, in which age was categorized as below 60 years compared with 60 years or older, younger age was a significant risk factor for POP recurrence after surgery [22, 24]. In 2 studies in which age was a continuous variable and in 1 study in which age was categorized as older than 70 years compared with 70 years or younger, no significant associations were found [23, 25, 26]. With regard to family history, 1 study found a significant association while another found no significant association [24, 25]. Menopausal status was not significantly associated with POP recurrence [22, 23].

#### Comorbidity

Constipation and pulmonary disease were not significantly associated with primary POP [13, 15, 21]. Urge and mixed urinary incontinence showed a significant association, while urinary incontinence surgery, stress urinary incontinence, and other forms of urinary incontinence were not significantly associated with primary POP [6, 21]. Diabetes mellitus, chronic illness, hysterectomy status, previous hernia surgery, and POP in pregnancy were examined once and only diabetes mellitus was significantly associated with primary POP [13, 15, 16, 21]. Owing to a lack of confirmation, no firm conclusions can be drawn.

Regarding POP recurrence, previous pelvic floor surgery and any preoperative urinary incontinence showed inconsistent results [22, 25, 26]. Constipation, pulmonary disease, and previous hysterectomy were not significant risk factors [22–26]. Incomplete bladder emptying, fecal incontinence, diabetes mellitus, and abdominal hernias were only investigated once and no significant associations were found [22, 24, 26]. Owing to a lack of confirmation, no firm conclusions can be drawn.

#### Social factors

Having less education was a significant risk factor for primary POP in 2 out of 4 studies, while occupation was not significantly related [6, 13, 15, 16, 20]. Income was only investigated once [13]. Women with a medium income were less likely to have POP compared with women with a high income, while the number of women with POP in the low income group was not significantly different from the number of women in the high income group.

The relation between social factors and POP recurrence was not evaluated in the 5 articles selected.

#### Pelvic floor factors

With regard to primary POP, levator defects and the genital hiatus on transperineal ultrasound were investigated as risk factors in 1 article [14]. Both a unilateral and a bilateral avulsion compared with no avulsion were significant risk factors for primary POP. An increased hiatal area on Valsalva was also associated with primary POP. For POP recurrence, levator defects, the site of most advanced prolapse, the genital hiatus on pelvic floor examination, and levator muscle contraction on pelvic floor examination were examined in 1 report, and only levator defects were significantly associated with POP recurrence [22–25]. Because of a lack of confirmative studies, no clear conclusion can be drawn.

#### Surgical factors

In 4 studies, preoperative stage 3 or 4 was a significant risk factor for POP recurrence after surgery [22–25]. Only the study in which preoperative stage 4 was compared with a preoperative stage of less than 4 found no significant association [26]. The number of sites involved preoperatively and the surgeon’s experience were not significant risk factors for POP recurrence [22, 24–26]. Concomitant surgery was examined in 1 article and a sacrospinous fixation was a significant risk factor for POP recurrence [25].

## Discussion

This systematic review provides an overview of the risk factors affecting the development of POP and POP recurrence after native tissue repair, investigated in cohort studies and cross-sectional studies. With regard to primary POP, parity, vaginal delivery, age, and BMI were the most important risk factors. Regarding POP recurrence, only preoperative stage was a confirmed risk factor.

The differences between risk factors for primary POP and POP recurrence might be explained by the differences in population. In studies concerning POP recurrence, only women with a primary POP are included; thus, this is a selected group of women. For instance, higher age was a risk factor for primary POP. If a woman obtained POP at a younger age, she might be more prone to POP recurrence after surgery than an older woman with POP, because of hereditary factors or connective tissue weakness. Indeed, the studies investigating the association between age and POP recurrence showed conflicting results. Perhaps the association between age and POP recurrence is not linear but parabolic, with both younger age and higher age being risk factors for POP recurrence. This is difficult to prove, but could explain the conflicting results. Other causes of the differences in confirmed risk factors for primary POP and POP recurrence might have been the smaller number of studies and the smaller sample sizes in the evaluation of risk factors for POP recurrence.

In the prevention of primary POP, BMI was the only modifiable risk factor. Theoretically, parity and vaginal delivery are also modifiable, but in obstetric care future POP seldom plays a role in considerations. With regard to preoperative counselling, only preoperative stage was a confirmed risk factor in the estimation of the chance of POP recurrence. The role of other patients' or surgeons’ characteristics was not confirmed.

There were several strengths and limitations of this review. The search was thorough and systematic. Two reviewers independently carried out the study selection and data extraction to minimize errors. Potential risk factors for both primary POP and POP recurrence were studied. We extracted the results of the multivariate analyses; thus, the reported effects were adjusted for potentially confounding variables. Studies with follow-up after surgery of less than 1 year were excluded to avoid bias due to surgical failures, which represents a different phenomenon than POP recurrence.

Only studies situated in western developed countries were included, because the population in developing countries may differ from that in developed countries. It has been stated that the prevalence of symptomatic POP among women in developing countries is higher than among women in developed countries, owing to early childbearing, high parity, low birth spacing, early return to work after delivery, poor birthing practices, frequent heavy lifting, and malnutrition [27]. Many women do not seek medical attention because of embarrassment, social taboos, fear of abandonment, knowledge deficit, lack of resources, and lack of access to trained personnel [28, 29].

Recurrence of POP was defined as anatomical recurrence after native tissue repair, i.e., without the use of mesh materials. Native tissue repair is the standard method of POP surgery, while the use of mesh in POP surgery has become controversial [30]. It is stated that the use of mesh should be reserved for high-risk individuals in whom the benefit of the use of mesh may justify the risks, such as individuals with recurrent POP [31]. The population in studies on POP recurrence after mesh surgery often consists of a selected, high-risk group of women, which cannot be compared with the population in studies on POP recurrence after native tissue repair.

Systematic reviews of prognostic studies are complicated by several issues, which have been well described by Altman [32]. Two major concerns are the quality of the primary studies and the possibility of publication bias. Although there is abundant literature to help researchers perform this type of research, there are still no widely agreed guidelines for assessing the quality of prognostic studies and there is no standard approach to building a multivariate prediction model [33]. Clear guidelines on the assessment of the quality of this type of study would be helpful.

Because of the enormous amount of available articles and variables studied on this subject, we were forced to select the papers providing the strongest evidence. We decided to exclude case–control studies because they are more prone to selection bias and often contain a smaller sample size than cohort or cross-sectional studies. Risk factors that have only been examined in case–control studies, such as collagen and matrix metalloproteinase polymorphisms, have been missed owing to this strategy.

Even after exclusion of case–control studies there was heterogeneity among the available studies. For example, the definitions of primary POP and the definitions of risk factors varied widely between studies, diverse covariates were used in multivariate analyses, and in the studies on POP recurrence there was diversity among the surgery performed. Because of this heterogeneity, it was not possible to perform a meta-analysis to pool the available results into reliable risk ratios. For uniformity, only articles were included with a definition of POP based on anatomical references such as the hymenal remnants or POPQ stage 2. POP recurrence was defined as anatomical recurrence after surgery, but this does not equate to recurrence or persistence of symptoms, which would have been a more patient-centered outcome [34]. Many women who may be categorized as “anatomical failures“ are, in fact, satisfied with their postsurgical results [35]. The problem with studies using only subjective findings for the definition of POP recurrence is that it is not possible to differentiate between the recurrence of POP in the same operated vaginal compartment and that in a different one [11]. That is why only studies in which pelvic floor examination was performed were included in this review. Uniformly accepted criteria for the definition of a successful POP operation are still lacking [36].

Furthermore, there are inconsistencies among studies as to whether a potential risk factor was indeed significantly associated with the primary outcome. Some potential risk factors were even protective against the primary outcome in one study, while they were a risk factor for that same outcome in another study. This made it difficult to come to conclusions. That is why we confirmed as risk factors only those that were significantly associated with POP or POP recurrence in at least two studies. Consequently, risk factors that have only been studied once and were significantly associated with POP or POP recurrence were not described as confirmed risk factors.

In conclusion, this systematic review showed that parity, vaginal delivery, age, and BMI were confirmed risk factors for the development of POP and that preoperative stage was a confirmed risk factor for POP recurrence after native tissue repair in western developed countries.

## Appendix A1: full PubMed literature search terms

((((pelvic prolapse[tiab] OR pelvic prolapses[tiab] OR ((((prolapse[tiab] OR prolapses[tiab]) AND (urogenital[tiab] OR genital[tiab] OR vaginal[tiab] OR pelvic organ[tiab]) OR cystocele[tiab] OR cystoceles[tiab] OR urinary bladder prolapse[tiab] OR urinary bladder prolapses[tiab])) OR rectocele OR rectoceles OR ("Pelvic Organ Prolapse"[Mesh])))) AND ((((("Recurrence"[Mesh])) OR (recurrence[tiab] OR recurrences[tiab] OR recurrent[tiab] OR relapse[tiab] OR relapses[tiab]))) OR (relapsing[tiab])))) OR ((("Risk Factors"[Mesh] OR risk[tiab] OR risks[tiab] OR "risk factor"[tiab] OR "risk factors"[tiab] OR “predict”[tiab] OR “predicts”[tiab] OR “prediction”[tiab] OR “predictions”[tiab] OR “predictive”[tiab] OR “predicting”[tiab])) AND (("Pelvic Organ Prolapse"[Mesh]) OR ((prolapse[tiab] OR prolapses[tiab]) AND (urogenital[tiab] OR genital[tiab] OR vaginal[tiab] OR pelvic organ[tiab] OR pelvic[tiab]) OR cystocele[tiab] OR cystoceles[tiab] OR urinary bladder prolapse[tiab] OR urinary bladder prolapses[tiab])))

## Appendix A2: full Embase literature search terms

((((pelvic prolapse[tiab] OR pelvic prolapses[tiab] OR ((((prolapse[tiab] OR prolapses[tiab]) AND (urogenital[tiab] OR genital[tiab] OR vaginal[tiab] OR pelvic organ[tiab]) OR cystocele[tiab] OR cystoceles[tiab] OR urinary bladder prolapse[tiab] OR urinary bladder prolapses[tiab])) OR rectocele OR rectoceles OR ("Pelvic Organ Prolapse"[Mesh])))) AND ((((("Recurrence"[Mesh])) OR (recurrence[tiab] OR recurrences[tiab] OR recurrent[tiab] OR relapse[tiab] OR relapses[tiab]))) OR (relapsing[tiab])))) OR ((("Risk Factors"[Mesh] OR risk[tiab] OR risks[tiab] OR "risk factor"[tiab] OR "risk factors"[tiab] OR “predict”[tiab] OR “predicts”[tiab] OR “prediction”[tiab] OR “predictions”[tiab] OR “predictive”[tiab] OR “predicting”[tiab])) AND (("Pelvic Organ Prolapse"[Mesh]) OR ((prolapse[tiab] OR prolapses[tiab]) AND (urogenital[tiab] OR genital[tiab] OR vaginal[tiab] OR pelvic organ[tiab] OR pelvic[tiab]) OR cystocele[tiab] OR cystoceles[tiab] OR urinary bladder prolapse[tiab] OR urinary bladder prolapses[tiab])))
